# Sex-Specific Effects of Organophosphate Diazinon on the Gut Microbiome and Its Metabolic Functions

**DOI:** 10.1289/EHP202

**Published:** 2016-05-20

**Authors:** Bei Gao, Xiaoming Bian, Ridwan Mahbub, Kun Lu

**Affiliations:** Department of Environmental Health Science, University of Georgia, Athens, Georgia, USA

## Abstract

**Background:**

There is growing recognition of the significance of the gut microbiome to human health, and the association between a perturbed gut microbiome with human diseases has been established. Previous studies also show the role of environmental toxicants in perturbing the gut microbiome and its metabolic functions. The wide agricultural use of diazinon, an organophosphate insecticide, has raised serious environmental health concerns since it is a potent neurotoxicant. With studies demonstrating the presence of a microbiome–gut–brain axis, it is possible that gut microbiome perturbation may also contribute to diazinon toxicity.

**Objectives:**

We investigated the impact of diazinon exposure on the gut microbiome composition and its metabolic functions in C57BL/6 mice.

**Methods:**

We used a combination of 16S rRNA gene sequencing, metagenomics sequencing, and mass spectrometry–based metabolomics profiling in a mouse model to examine the functional impact of diazinon on the gut microbiome.

**Results:**

16S rRNA gene sequencing revealed that diazinon exposure significantly perturbed the gut microbiome, and metagenomic sequencing found that diazinon exposure altered the functional metagenome. Moreover, metabolomics profiling revealed an altered metabolic profile arising from exposure. Of particular significance, these changes were more pronounced for male mice than for female mice.

**Conclusions:**

Diazinon exposure perturbed the gut microbiome community structure, functional metagenome, and associated metabolic profiles in a sex-specific manner. These findings may provide novel insights regarding perturbations of the gut microbiome and its functions as a potential new mechanism contributing to diazinon neurotoxicity and, in particular, its sex-selective effects.

**Citation:**

Gao B, Bian X, Mahbub R, Lu K. 2017. Sex-specific effects of organophosphate diazinon on the gut microbiome and its metabolic functions. Environ Health Perspect 125:198–206; http://dx.doi.org/10.1289/EHP202

## Introduction

The gut microbiome plays a profound role in metabolic processing, energy production, immune and cognitive development and epithelial homeostasis (Nicholson et al. 2012). Changes to the gut microbiome are highly related to host physiological conditions and pathophysiological parameters and are associated with a long list of diseases including obesity, diabetes, cardiovascular disease, inflammatory bowel disease, colon cancer, liver cirrhosis, neuropathology, and other diseases (Cani et al. 2013; Qin et al. 2014). The gut microbiome not only induces effects localized in the gut, but also influences many distant organs. For example, the gut microbiome can affect human brain health via structural bacterial components, proteins, enzymes, hormones, or stimuli directly caused by the gut microbiome itself (Galland 2014). In particular, the gut microbiome can affect host physiology by the production of metabolites or the conversion of host compounds. For instance, when the gut microbiome ferments complex carbohydrates and plant polysaccharides that cannot be digested by human enzymes, short chain fatty acids (SCFAs) are produced. SCFAs are energy substrates for peripheral tissues and the colonic epithelium, and the type and quantity of SCFAs are affected by the composition of gut microbiome (Turnbaugh et al. 2006). Likewise, the gut microbiome biotransforms a number of host compounds. For example, the gut microbiome modifies primary bile acids to secondary bile acids (Ridlon et al. 2006). Bile acids not only facilitate the digestion and absorption of fat and fat-soluble vitamins and cholesterol, they also serve as signaling molecules correlated with the complex nuclear receptor network to affect key host transcription programs (Degirolamo et al. 2011). Given the important roles of the gut microbiome and gut microbiome-related metabolites in regulating normal physiological functions, environmental toxicant-induced functional perturbations of the gut microbiome may lead to or exacerbate their toxicity.

A few recent studies have documented the effects of environmental chemicals on gut microbiome community structures (Choi et al. 2013; Narrowe et al. 2015); however, the functional impacts of environmental chemicals on the gut microbiome are not well established yet. In this regard, we recently demonstrated that arsenic exposure not only alters the gut microbiome community at the abundance level but also disturbs its metabolic profiles (Lu et al. 2014a). We also found that gut microbiome phenotypes, driven by host genetics or influenced by bacterial infection, could affect arsenic biotransformation (Lu et al. 2013, 2014b), highlighting individual variations in the gut microbiome that could modulate the toxicity of environmental chemicals. It is noteworthy that the composition and temporal variability of the human microbiome is actually highly personalized (Flores et al. 2014). Certainly, a better understanding of the interplay between environmental exposure and the gut microbiome should provide new mechanistic insights into environmental chemical-induced human disease and related susceptibility.

Diazinon is an organophosphate insecticide widely used in conventional agriculture, and has been detected in ground water, agriculture wells, drinking water wells, and monitoring wells, resulting in human exposure (Aggarwal et al. 2013). A study from the U.S. Environmental Protection Agency (EPA) found the presence of diazinon metabolites in the urine of children (Egeghy et al. 2011). Thus, the prevalence and toxicity of diazinon has raised serious public health concern. In addition to its major neurotoxicity, diazinon is associated with oxidative stress (Čolović et al. 2015), cardiotoxicity (Zafiropoulos et al. 2014), and vascular toxicity (Razavi et al. 2014). Although it is generally accepted that diazinon acts through the inhibition of acetylcholinesterase (AChE) in both the central and peripheral nervous system (Casida and Quistad 2004), several studies show that diazinon induced neurotoxicity in animals at concentrations lower than the threshold for AChE inhibition, suggesting other modes of action may play a role in diazinon neurotoxicity (Slotkin et al. 2008; Timofeeva et al. 2008).

Given the indispensable and complex roles of the gut microbiome in regulating normal physiological functions, the interaction between diazinon and the gut microbiome may play a role in its toxicity; However, there is virtually no information about how diazinon and the gut microbiome interplay. Of particular interest, previous studies reported sex-selective neurotoxicity induced by diazinon, with more pronounced effects being observed in male rats (Roegge et al. 2008; Slotkin et al. 2008; Timofeeva et al. 2008). Meanwhile, distinct features of male and female microbiomes in mice have been documented (Markle et al. 2013), raising the possibility that environmental toxicants may perturb the microbiome and its associated functions in a sex-selective manner. Therefore, this study was designed to address three questions: *a*) whether diazinon exposure alters the gut microbiome community structure; *b*) whether diazinon exposure changes the metabolic functions of the gut microbiome; and *c*) whether these effects are sex specific. To address these questions, we applied omics-based systems-level approaches, including 16S rRNA gene sequencing, shotgun metagenomics sequencing, and gas chromatography-mass spectrometry (GC-MS) metabolomics profiling to probe functional interactions between diazinon exposure and the gut microbiome. To our knowledge, this study provides the first evidence to demonstrate the functional interactions between the gut microbiome and diazinon exposure.

## Materials and Methods

### Animals and Exposure

Twenty specific pathogen-free C57BL/6 mice (~ 7 weeks old), previously co-housed in the same cages over the weaning period, were purchased from Jackson Laboratory (Bar Harbor, ME) and housed in the University of Georgia animal facility for a week before the start of experimentation, where they were allowed to consume tap water *ad libitum*. Before and throughout the experimental period, mice were housed under environmental conditions of 22°C, 40–70% humidity, and a 12:12 hr light:dark cycle and were provided with standard pelleted rodent diet. At the start of experimentation, mice were randomly assigned to either a control (five male and five female mice) or diazinon-treated group (five male and five female mice). Diazinon was administered to mice in drinking water at a concentration of 4 mg/L for a study period of 13 weeks; This low dose was equivalent to those used in previous studies (Roegge et al. 2008), which did not elicit discernible AChE inhibition. Control mice received water alone. Body weight was measured weekly. The animal protocol was approved by the University of Georgia Institutional Animal Care and Use Committee. The animals were treated humanely and with regard for alleviation of suffering.

### 16S rRNA Gene Sequencing

16S rRNA gene sequencing was performed as described previously (Lu et al. 2014a). Briefly, total DNA was extracted from fecal pellets collected during necropsy using PowerSoil® DNA isolation kit according to the manufacturer’s instructions. For 16S gene sequencing, DNA was amplified using 515F and 806R primers (Caporaso et al. 2012) to target the V4 regions of 16S rRNA gene, followed by normalization and barcoding. The resulting DNA were pooled, quantified by Qubit 2.0 Fluorometer and sequenced at the Georgia Genomics Facility using an Illumina MiSeq (500 cycles v2 kit). Paired-reads were assembled in Geneious (Biomatters, Auckland, New Zealand), followed by trimming end with error probability of 0.01 as initial quality filtering. Quantitative Insights into Microbial Ecology (QIIME) software (version 1.9.1; http://qiime.org/) was used. De novo operational taxonomic unit (OTU) picking was used and OTUs were chosen with a threshold of 97% sequence similarity. A representative sequence from each OTU was selected for taxonomic assignment according to Greengenes database (version 13_5; http://greengenes.lbl.gov/). By default, QIIME uses uclust consensus taxonomy classifier to assign taxonomy. Each representative 16S rRNA sequence was assigned at different levels, including phylum, class, order, family and genus. CloVR 1.0-RC4 was used with default pipelines and parameters. CloVR 16S pipeline employs several phologenetic tools, including QIIME, UCHIME, Mothur, and Metastats (Angiuoli et al. 2011).

### Metagenomics Sequencing

For metagenomics sequencing, DNA (10 ng/μL) was fragmented using the Bioruptor UCD-300 sonication device (Diagenode Inc., Denville, NJ), followed by sequencing library construction using the Kapa Hyper Prep Kit according to the manufacturer’s instructions. The resulting DNA was pooled, quantified, and sequenced at the Georgia Genomics Facility using an Illumina NextSeq High Output Flow Cell. The raw fastq files were imported into the MG-RAST: Metagenomics Rapid Annotation using Subsystems Technology (version 3.5; MG-RAST ID 12453) (Meyer et al. 2008). Sequences were assigned to M5NR Subsystems database for functional analysis with maximum e-Value cutoff 10^–5^, 75% minimum identity cutoff, and minimum alignment length cutoff of 35.

### Metabolomics Analysis

Metabolites were isolated using the method described previously with some modifications (Lu et al. 2014a). To isolate metabolites from feces, two fecal pellets (~ 20 mg) were vortexed with 1ml of methanol, chloroform, and water solution (2:2:1) for 20 min, followed by centrifugation at 1,000 × g for 15 min. The resultant upper phase and lower phases were transferred to a flat-bottom HPLC vial and were dried for 4 hr in a SpeedVac, followed by derivatization with methoxyamine-HCL and BSTFA. To isolate the metabolites from serum, cold methanol (80 μL) was added to 20 μL serum then vortexed at the highest speed for 1 min. The samples were incubated at 4°C for 20 min and then centrifuged for 10 min at 12,000 rpm. The supernatant was collected, dried in a SpeedVac, and followed by derivatization using methoxyamine-HCL and BSTFA. The samples were then injected into an Agilent GC-MS running at a full scan mode. The resultant data were processed with XCMS Online for peak picking, alignment, and extraction of peak intensities. The molecular features with significant changes (*p* < 0.05, fold change > 1.5) were identified in the National Institute of Standards and Technology (NIST) Standard Reference Database and searched against the Human Metabolome Database (version 3.0; www.hmdb.ca/) for their function and pathway information.

### Statistical Analysis of Data

The difference in the gut microbiome composition was assessed using a nonparametric test via Metastats software (http://metastats.cbcb.umd.edu/) as described previously (White et al. 2009). Principle coordinate analysis (PCoA) was used to compare the gut microbiome profiles between control and treatment samples, which examine the beta diversity difference based on the UniFrac distance metric (Lozupone and Knight 2005). To initially profile individual metabolite differences between control and treatment groups, a two-tailed Welch’s *t*-test was used (*p* < 0.05), as described elsewhere (Lu et al. 2014a; Wikoff et al. 2009). Also, partial least squares discriminant analysis (PLS-DA) for the control and treatment groups was performed, followed by molecular feature selection for metabolite identification based on Variable Importance in Projection (VIP) scores (VIP > 1.8), as described previously (Chan et al. 2011; Cui et al. 2013). The VIP scores estimate the importance of each variable in the projection used in the PLS model. Finally, heat maps were generated using hierarchical clustering algorithm to visualize the metagenomic function and metabolite differences within the dataset.

## Results

### Diazinon-Induced Changes of the Gut Microbiome Community Structures

**Figure 1:**
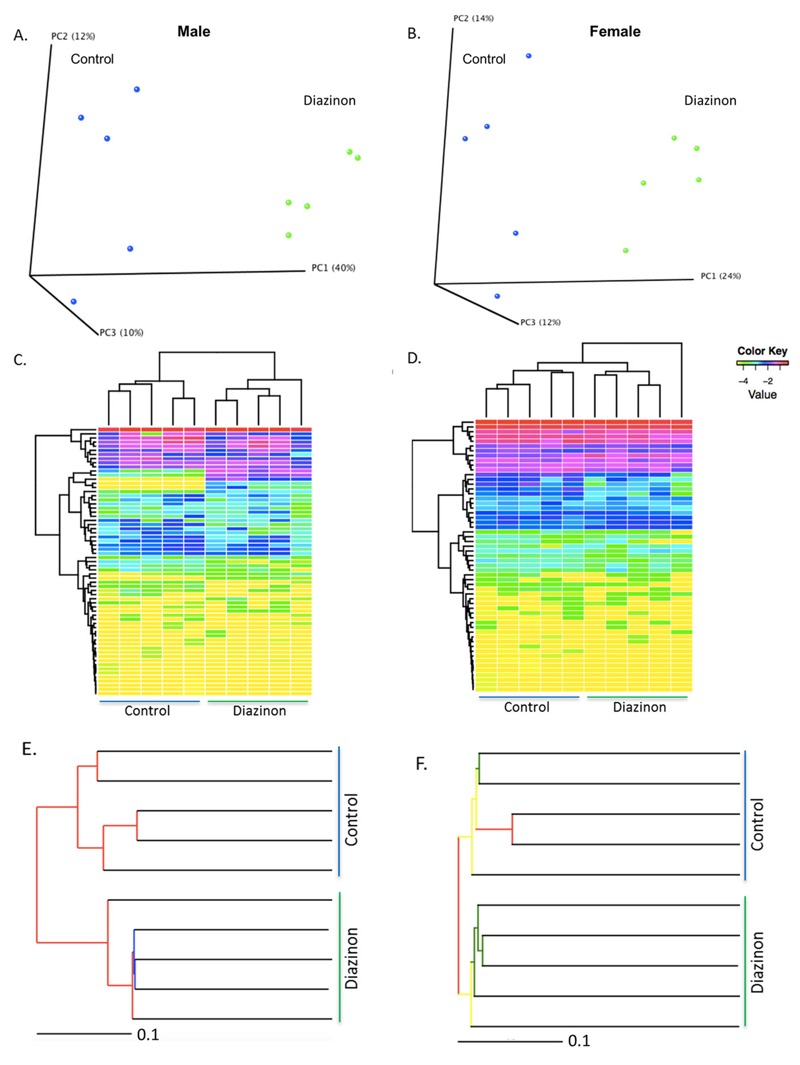
Diazinon exposure altered the gut microbiome community structures in both (*A*) male and (*B*) female animals, as illustrated by the 3D PCoA plots based on the UniFrac distance metric and beta diversity (Red: controls; Blue: diazinon-treated animals); the hierarchical clustering analysis reveals that diazinon-treated mice and controls generally cluster in their own groups using the gut bacteria analyzed at the genus level (*C*: males; *D*: females); the jackknifed beta diversity via the unweighted pair group method with arithmetic mean (UPGMA) demonstrated that control and diazinon-treated male and female mice have distinct clustering patterns within each group (*E*: males; *F*: females), with the UPGMA distance tree constructed at a distance of 0.1.

A total of 421,813 good quality sequence reads were generated. All reads with < 97% similarity with the known organisms in Greengenes database were not considered for further analysis. Representative sequences for each OTU were assigned to multiple taxonomic levels (phylum to genus) according to the Greengenes database. 3D PCoA plots (version 1.9.1; QIIME) (Figure 1) showed the gut microbiome community structures at the genus level were readily differentiated between controls and diazinon-treated mice. In males, 40%, 12%, and 10% of the variation was explained by principle component (PC) 1, PC2, and PC3, respectively. In female mice, 24%, 14%, and 12% of the variation was explained by PC1, PC2, and PC3, respectively; these results suggest that male mice were better separated than female mice. Consistent with the PCoA plot, hierarchical clustering analysis (Figure 1C,D) and the jackknifed beta diversity via the unweighted pair group method with arithmetic mean (UPGMA) (Figure 1E,F) demonstrated that control and treated male and female mice typically clustered in their own groups.

### Perturbed Gut Bacteria in Male and Female Mice

**Figure 2:**
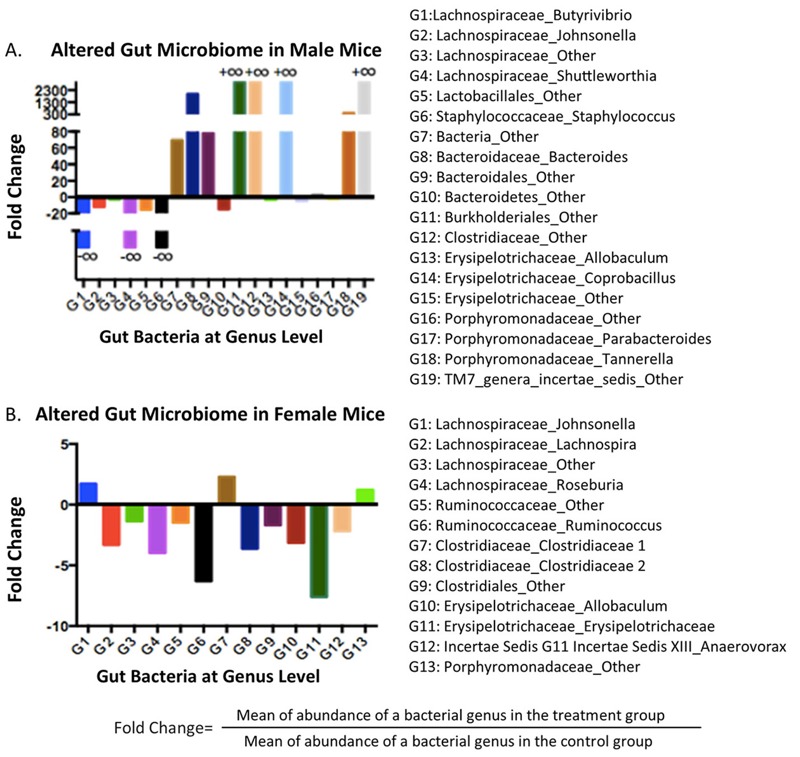
Diazinon exposure altered specific bacterial genera in a highly sex-specific manner, with stronger responses being observed in (*A*) males than in (*B*)**females. (+∞: bacterial genus was only observed after treatment; –∞: bacterial genus was completely inhibited by exposure; the fold change illustrates the abundance alteration of a bacterial component between the treatment and control groups.) Note: Only the fold changes for statistically significantly altered bacterial genera are presented here.

Of particular interest, diazinon induced sex-specific effects on the gut microbiome, as evidenced by several perturbed gut bacterial genera and different patterns of changes in male and female mice after a 13-week exposure (illustrated in Figure 2; see also Tables S1 and S2 for individual variations of all bacteria genera). For example, a total of 19 bacterial genera were altered in male mice, with 9 and 10 being increased and decreased, respectively, while a total of 13 bacterial genera were changed in female mice, with the majority being reduced. More importantly, the taxonomy classifications of perturbed bacterial genera were very different in male and female animals. Moreover, the fold changes of specific genera are much higher for male mice than for females. In particular, several bacteria genera in males, such as G8 (*Bacteroidaceae_Bacteroides*), G11(*Burkholderiales_Other*), G12 (*Clostridiaceae_Other*), and G14 (*Erysipelotrichaceae_Coprobacillus*) were only observed after diazinon treatment, while G1 (*Lachnospiraceae_Butyrivibrio*), G4 (*Lachnospiraceae_Shuttleworthia*), and G6 (*Staphylococcaceae_Staphylococcus*) were completely inhibited in males by exposure. In addition, diazinon had a sex-selective effect even for the same bacterial genus. For example, *Lachnospiraceae_Johnsonella* reduced –16-fold in male mice after exposure, but increased +1.8-fold in female animals. The sex-dependent gut microbiome background may largely contribute to sex-selective responses to diazinon exposure, as our data illustrated that the gut microbiome profiles were significantly different between male and female mice (see Figure S1), consistent with the previous report regarding sex-dependence of microbiome features (Markle et al. 2013).

### Diazinon-Induced Metagenome Changes

**Figure 3:**
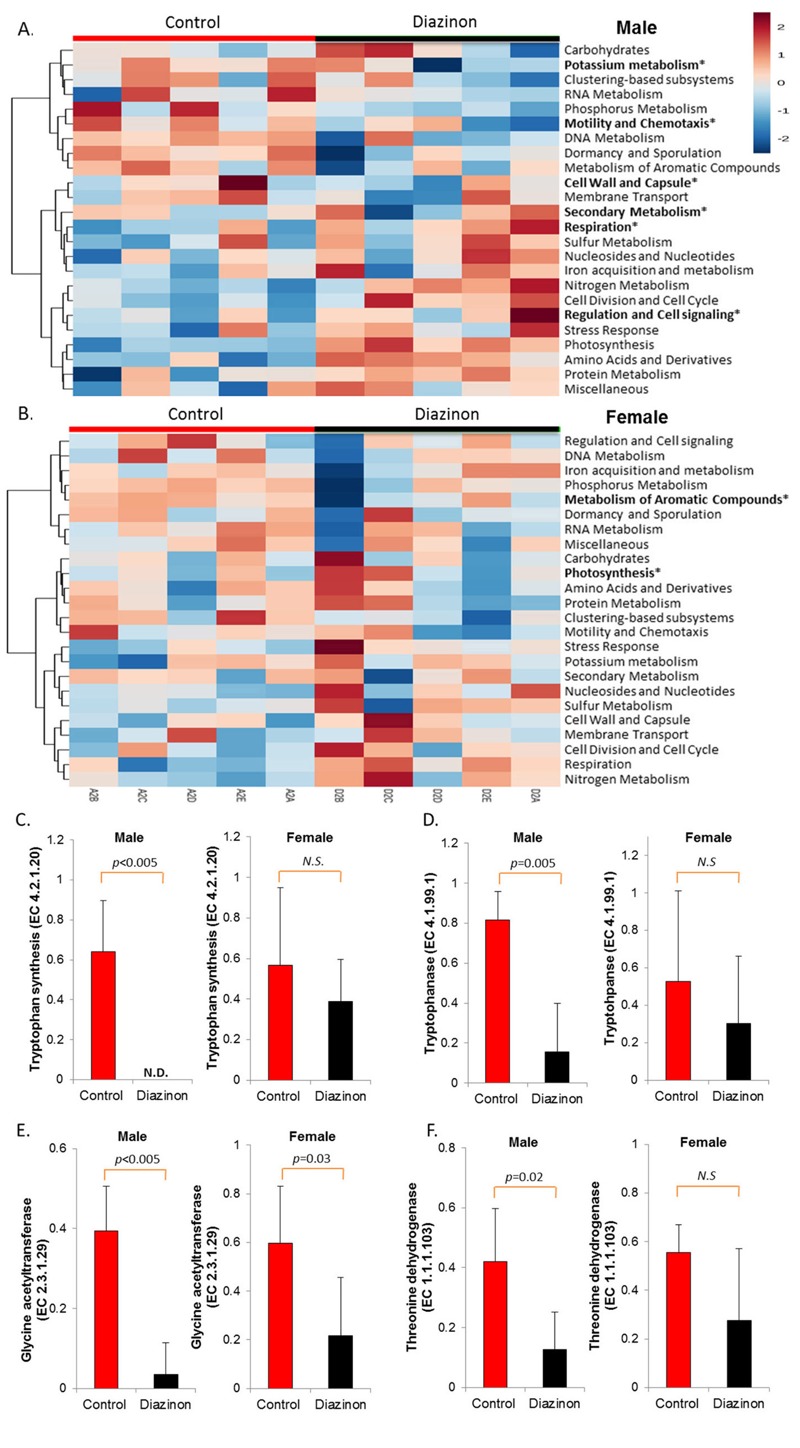
Sex-selective effects of diazinon exposure on modulating functional metagenome, as illustrated by the heat map constructed with the results from the level 1 analysis of subsystem for (*A*) males and (*B*) females. Analysis at the function level of subsystem demonstrated sex-specific perturbations of several key genes involved in the synthesis pathways of neurotransmitters and related metabolites, with more significant downregulation being observed in male animals (*C* and *D*: tryptophan and indole pathway; *E* and *F*: glycine synthesis pathway). Note: N.D., nondetectable; N.S., no statistical significance. *p < 0.05.

In addition to taxonomy, metagenomic analysis provides a functional profile of the whole bacterial genome, among which many genes are involved in specific metabolic pathways or functions that are related to host metabolism. As shown in Figure 3, the hierarchical clustering heat map, constructed using the results from the level 1 analysis of subsystem illustrated that control and diazinon-treated mice had sex-specific changes of metagenomic functions. Specifically, in male mice, secondary metabolism, potassium metabolism, regulation and cell signaling, motility and chemotaxis, cell wall and capsule, and respiration were significantly perturbed (*p* < 0.05), while only photosynthesis and metabolism of aromatic compounds were altered in female mice (*p* < 0.05). Likewise, analysis performed at the level 2, level 3, and function level of subsystem also indicated that diazinon exposure induced sex-specific metagenomic perturbations, with greater and differential effects being found in male than in female mice (see Figures S2 and S3). At the function level, 391 and 190 classified functions with *p* < 0.05 were found in male and female mice, respectively. In the context of neurotoxicity, it is noteworthy that several critical bacterial genes involved in synthesis and regulation of neurotransmitters or related metabolites were more significantly perturbed in male than female mice, as illustrated in Figure 3C–F.

### Diazinon-Induced Changes in the Metabolic Profile of the Gut Microbiome

**Figure 4:**
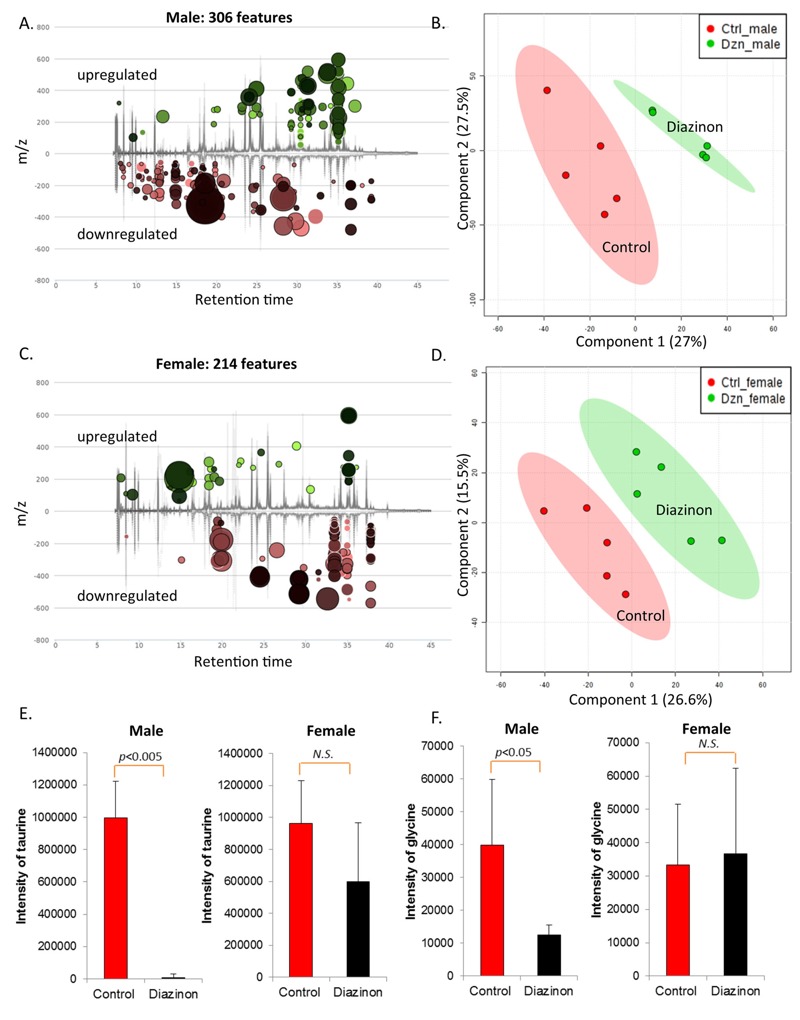
Diazinon exposure perturbed the metabolic profiles of fecal samples of mice, with 306 and 214 molecular features being significantly changed in (*A*) male and in (*C*) female animals, respectively (fold change > 1.5 and *p* < 0.05). Controls were separated from diazinon-treated mice in fecal metabolite profiles by PLS-DA (*B*: males; *D*: females). Two major neurotransmitters, taurine and glycine, were more significantly downregulated in male than female mice (*E*: taurine; *F*: glycine). Note: N.S., no statistical significance.

Figure 4 shows that diazinon exposure perturbed the metabolic profiles of the gut microbiome in both male and female mice after a 13-week exposure, but male mice had relatively stronger responses following exposure. Specifically, 306 and 214 molecular features with *p* < 0.05 and fold changes > 1.5 were found in male and female mice, respectively. This observation was consistent with 16S and metagenomics analysis, as described previously. The control and diazinon-treated groups could be differentiated readily in both male and female mice using metabolite features (Figure 4B,D), with an excellent separation of the control and diazinon-treated animals using the first two components of PLS-DA. Clear separations of metabolite profiles between control and diazinon-treated animals were also observed for serum samples (see Figure S4). Molecular features with a *p*-value less than 0.05 and fold changes greater than 1.5 were filtered using the VIP scores (VIP > 1.8, see Figure S5) for further identification and metabolites with diverse structures were identified (see Tables S3 and S4). In particular, two neurotransmitters, taurine and glycine (Figure 4E,F), were more significantly downregulated in male than female mice, which is consistent with sex-specific modulation of metagenome and key genes involved in the synthesis and regulation of neurotransmitters, as illustrated in Figure 3.

## Discussion

We used integrative methods combining 16S rRNA gene sequencing, shotgun metagenomics sequencing and metabolomics profiling to study the effect of diazinon exposure on the gut microbiome and its metabolic processes. We have demonstrated that diazinon exposure changed gut microbiome taxonomic compositions, functional metagenome, and metabolic profiles in C57BL6 mice, highlighting the functional impact of diazinon exposure on the gut microbiome. In particular, diazinon perturbed the gut microbiome and its metabolic functions in a sex-specific manner, with more pronounced responses being observed for male than female mice. This sex-dependent sensitivity may be linked to differential neurotoxicity of diazinon in male and female animals, which has been reported in previous studies with few mechanisms being identified yet (Roegge et al. 2008; Slotkin et al. 2008; Timofeeva et al. 2008).

Diazinon exposure significantly altered the community structures of the gut microbiome of mice in a sex-specific manner (Figures 1 and 2). We have identified a number of bacterial genera perturbed by diazinon exposure. In general, several genera of *Lachnospiraceae* family decreased in both male and female animals. *Lachnospiraceae* is an important bacterial family involved in the production of short chain fatty acids, and patients with depressive disorders have been reported to be associated with reduced *Lachnospiraceae* (Jiang et al. 2015). Meanwhile, the prevalence of several potentially pathogenic bacteria was only observed in treated male mice. For instance, the *Burkholderiales* order comprises several families of gram-negative bacteria, which have been implicated in human diseases including respiratory infections, chronic granulomatous disease and inflammatory bowel disease (Sim et al. 2010). Likewise, *Erysipelotrichaceae_Coprobacillus* was reported to be significantly different between irritable bowel syndrome patients and healthy controls (Lee and Bak 2011). In females, several families, such as *Lachnospiraceae, Ruminococcaceae, Clostridiaceae* and *Erysipelotrichaceae*, decreased after exposure to diazinon. These families and associated genera are key components of the gut microbiome and have important physiological functions to the host. In addition, the gut microbiome is highly related with energy metabolism and harvest in the host (Turnbaugh et al. 2006), with an increased ratio of *Firmicutes/Bacteroidetes* being associated with obese individuals. Our 16S rRNA gene sequencing results showed that at the phylum level, *Bacteroidetes* increased by 1.8-fold while *Firmicutes* decreased by 1.8-fold in diazinon-treated male mice from controls, suggesting the energy metabolism and harvest could be inhibited by treatment in male mice. In fact, the body weight gain of male mice was significantly decreased (7.6 g and 4.3 g for controls and diazinon-treated males, respectively, *p* < 0.05) at the end of the 13-week exposure; whereas, no significant effects of diazinon on body weight was observed in female animals (see Figure S6). These results again demonstrated different responses to diazinon exposure in a sex-specific manner.

Consistent with the changes of the gut microbiome community structures, the functional metagenome was also significantly altered, with a number of key enzymes being altered by diazinon. In particular, several key genes involved in synthesis of neurotransmitters and related metabolites are significantly perturbed, especially in male animals. For example, tryptophan synthase beta chain was remarkably downregulated in treated male mice (*p* < 0.005), whereas no statistically significant difference was observed in female animals (Figure 3C). Likewise, tryptophanase was also significantly downregulated in male animals but not in females (Figure 3D). Tryptophanase converts tryptophan to indole, a key substrate for bacteria to synthesize other indole-containing metabolites. Thus, reduced tryptophan synthase and tryptophanse may lead to perturbed synthesis and tissue distribution of tryptophan-related and indole-containing metabolites such as serotonin and other neurotransmitters. In agreement with this potential regulation mechanism, we previously reported that a number of indole-containing compounds including the metabolite of serotonin and tryptophan-related metabolic pathways were significantly altered in an animal model with an altered gut microbiome arising from bacterial infection (Lu et al. 2012). Consistently, a recent study has demonstrated that the microbiota plays a critical role in regulating host serotonin, an indole-containing neurotransmitter (Yano et al. 2015). Likewise, two key enzymes that acts in concert to produce glycine from threonine, glycine acetyltransferase and threonine dehydrogenase were also more significantly downregulated in male than female mice (Figure 3E,F). Consistent with this metagenomic change, glycine was found to be reduced in male animals (–3.2-fold), but not in female ones (Figure 4F). Alteration of these well-established neurotransmitters and signaling molecules has been associated with neurotoxicity (Bjørling-Poulsen et al. 2008). Taken together, sex-specific changes of functional metagenome and related metabolic pathways may point to the potential role of the gut microbiome in eliciting different neurotoxicity in male and female animals.

We also found that the abundance of bile acids was significantly altered by diazinon exposure (see Tables S3 and S4). The gut microbiome modifies bile acids steroid core through a 7alpha-dehydroxylation reaction and modification of side chain through deconjugation by bile salt hydrolases to form secondary bile acids such as deoxycholate and lithocholate (Degirolamo et al. 2011). Bile salt hydrolase enzymes have been identified mainly in anaerobes of the genera *Bacteroides, Clostridium, Eubacterium, Lactobacillus,* and *Escherichia* and a small portion of bile acid transformation could also be conducted by aerobic bacteria such as *actinobacteria* and *proteobacteria* (Nicholson et al. 2012). Here, we observed several gut bacterial changes that are related to bile acid pathways. Specifically, *Bacteroidaceae_Bacteroides* significantly increased (> 2,000-fold, Figure 2), from which bile salt hydrolase has been identified. Likewise, *Proteobacteria*, which could perform bile acid transformation, was also increased (+15-fold, Figure 2). Consequently, a perturbed pool of bile acids are expected to occur. In fact, several metabolites related to bile acids metabolism were changed by diazinon. Lithocholic acid (LCA) level increased by 4-fold in diazinon-treated males compared to controls, accompanied by a 1.8-fold increase of cholesterol, the precursor of bile acids. LCA is a toxic hydrophobic secondary bile acid formed in the large intestine by bacterial 7alpha-dehydroxylation of chenodeoxycholic acid (CDCA) (Ridlon et al. 2006). The increase of this secondary bile acid indicated that deconjugation activity increased which may be related to the increase in bundance of *Bacteroides* and *Proteobacteria*. The increase of LCA in diazinon-treated animals is noteworthy, as a recent study showed that LCA feeding resulted in direct hepatotoxicity (Woolbright et al. 2014). Likewise, the increase of secondary bile acids in feces is considered a risk factor for colon cancer (Okazaki et al. 2012). LCA level in diazinon-treated female mice showed a similar trend with a 5-fold increase, but in contrast to male mice, the cholesterol level decreased by 2-fold in diazinon-treated female mice. In addition, 5beta-cholestanone, an oxidation product of 5beta-coprostanol formed via bio-hydrogenation of cholesterol, decreased by 5-fold in diazinon-treated female mice, which was not statistically significantly altered in male mice. Besides LCA, another secondary bile acid, deoxycholic acid, increased 3.6-fold in diazinon-treated female mice. Together, these data demonstrate that diazinon perturbed bile acid metabolism in both male and female animals, but the patterns and involved metabolites were sex dependent.

Of particular interest, we found that free taurine level decreased > 100-fold (Figure 4E) in the feces of diazinon-treated male mice, while no significant change was found in females. Taurine is conjugated to bile acids in liver and deconjugated in the intestine by several bacterial species. Previous studies showed an increase in taurine-conjugated bile acids in multiple body compartments in germ free and antibiotic treated rats and higher free taurine levels in the liver of germ free rats (Swann et al. 2011), highlighting the role of the gut microbiome in regulating taurine and bile acid pathways. The underlying mechanisms responsible for the modulation of fecal taurine abundance remain elusive; however, diazinon-induced gut microbiome perturbations may play a role in this process. In this study, *Staphylococcus* was completely inhibited in diazinon-treated male mice compared to controls. *Staphylococcus* was reported to be one of the most relevant bacterial genera that are correlated with the balance in taurine-conjugated bile acids in the bile acid and fecal flora correlation network (Martin et al. 2008). Interestingly, no significant change in taurine was observed in diazinon-treated female mice. Again, the difference in the regulation of taurine clearly indicates sex-specific effects of diazinon on the gut flora-related metabolic functions and metabolites.

As a well-established neurotoxicant, the major mode of action of diazinon is to inhibit AChE. Meanwhile, previous studies also suggest that other mechanisms could be involved in organophosphate pesticide-induced neurotoxicity (Casida and Quistad 2004; Slotkin et al. 2008). On the other hand, with the emerging concept of a microbiome gut–brain axis, studies are revealing how the gut microbiome is connected with nervous system disorders including autism, depression, anxiety, and stress, and how microbiome-related products could modulate these disorders (Foster and McVey Neufeld 2013). As demonstrated in this study, diazinon exposure significantly altered the gut microbiome and its associated metabolites, raising the possibility that perturbation of the gut microbiome contributes to the neurotoxicity of organophosphate pesticides. Consistent with our finding of sex-specific effects of diazinon on the gut microbiome, several previous studies found that diazinon induced differential outcomes in male and female animals (Foster and McVey Neufeld 2013; Roegge et al. 2008; Slotkin et al. 2008; Timofeeva et al. 2008). For example, neonatal diazinon exposure had lasting effects on emotional responses, as found by elevated plus maze test and novelty-suppressed feeding tests, with preferential effects on male animals (Roegge et al. 2008). Unlike the present study, neonatal animals were used previously; however, the reprograming effect of diazinon exposure on the gut microbiome in young animals may be more pronounced, because the establishment of the gut microflora is a temporal and sensitive process occurring after birth (Palmer et al. 2007). Future studies are needed to address age-related effects of diazinon exposure by examining the trajectory of microbiome establishment. It remains unclear how sex-specific effects occur following exposure to diazinon; however, perturbation of the gut microbiome and its key metabolic pathways may provide a clue. For example, taurine reduced more than 100-fold in the feces of male mice, while no significant difference was observed in female ones. Additionally, a previous study found that taurine in the brain significantly decreased in male animals after chronic intake of diazinon for 28 weeks (Rajendra et al. 1986). Meanwhile, taurine has multiple roles in the central nervous system, such as serving as a neurotransmitter, functioning as a trophic factor and regulating calcium transport and homeostasis (Wu and Prentice 2010). In particular, taurine has inhibitory effects on neurotransmission, like other neurotransmitters GABA and glycine (also downregulated in male mice only, Figure 4F) do (El-Idrissi and Trenkner 2004). Taurine modulates neuronal excitability via either direct enhancement of GABAergic function and/or indirect depression of glutamatergic neurotransmission (El-Idrissi and Trenkner 2004). In addition, taurine increases the activity of paraoxonase I, an enzyme that is involved in organophosphate metabolism and detoxification (Dirican et al. 2007). Thus, reduced taurine in males only may contribute to more pronounced neurotoxicity as observed in several previous studies (Roegge et al. 2008; Slotkin et al. 2008). Taurine also was reported to increase the number of somatostatin-positive neurons in the cortex and hippocampus, which ultimately enhances cognitive function (Levinskaya et al. 2006). Moreover, taurine supplement has been demonstrated to attenuate neuronal degeneration induced by organophosphate pesticides and heavy metals (Akande et al. 2014). Taken together, our data suggest that a perturbed gut microbiome and associated metabolic functions may point to a novel mode of action underlying diazinon-induced sex-specific effects. Clearly, further research is needed to shed light on the role of the gut microbiome and its associated key metabolites in diazinon neurotoxicity and its sex-selective effects.

There are several limitations of this study. Humanized gnotobiotic animals or mice with a standardized murine microbiome were not used, but they could serve as better models to reduce the individual variations of gut bacteria. Individual variations associated with non-standardized animals could significantly impair the statistical power for the detection of altered microbiome components and data comparison across studies. In addition, it has been demonstrated that the metabolism of diazinon plays a role in the sex-specific neurotoxicity (Jansen et al. 2009). It remains unknown whether the gut microbiome contributes to the sex-selective metabolism of organophosphates. Moreover, we have demonstrated that diazinon exposure induced sex-selective changes of multiple gut microbiome endpoints, however, the causative role of gut microbiome in sex-specific neurotoxicity of diazinon could not be established yet. Furthermore, we discovered several critical changes of functional metagenome and neurotransmitters in the gut, but molecular mechanism underlying the crosstalk between the gut microbiota and neurotoxicity need to be further elucidated. Likewise, the age-related and/or transgenerational effects of organophosphates on reprogramming the microbiome also need to be examined in the future. Answers to abovementioned questions all await future studies.

## Conclusion

In summary, we have demonstrated that diazinon perturbed the gut microbiome community structure, functional metagenome, and metabolic profiles of the gut microbiome in a sex-specific manner, with stronger responses being observed in male mice. These findings may provide novel understanding of the role of the gut microbiome in neurotoxicity of organophosphate pesticides. Perturbation of the gut microbiome and its associated metabolic functions may serve as a new mechanism underlying sex-selective effects of organophosphates. Future study is warranted to understand the role of the gut microbiome and its related key metabolites in modulating sex-selective toxicity of organophosphates and other relevant environmental chemicals.

## Supplemental Material

(900 KB) PDF
