# Supplementary material for: Sex-Specific Effects of Organophosphate Diazinon on the Gut Microbiome and Its Metabolic Functions
Source: Environ Health Perspect. 2016 May 20;125(2):198–206. doi: 10.1289/EHP202 (PMC5289904; doi:10.1289/EHP202)
Supplement: (900 KB) PDF [file EHP202.s001.acco.pdf]

Environ Health Perspect
DOI: 10.1289/EHP202

Note to readers with disabilities: *EHP* strives to ensure that all journal content is accessible to all readers. However, some figures and Supplemental Material published in *EHP* articles may not conform to 508 standards due to the complexity of the information being presented. If you need assistance accessing journal content, please contact ehp508@niehs.nih.gov. Our staff will work with you to assess and meet your accessibility needs within 3 working days.

Supplemental Material

Sex-Specific Effects of Organophosphate Diazinon on the Gut Microbiome and Its Metabolic Functions

Bei Gao, Xiaoming Bian, Ridwan Mahbub, and Kun Lu

Table of Contents

Table S1. Relative abundance of typical gut bacterial genera in the measurements for control and diazinon exposed male mice

Table S2. Relative abundance of typical gut bacterial genera in the measurements for control and diazinon exposed female mice

Table S3. Identified significantly changed fecal metabolites (fold change >1.5 p < 0.05 compared to controls) in male mice

Table S4. Identified significantly changed fecal metabolites (fold change >1.5 p < 0.05 compared to controls) in female mice

Figure S1. Distinct gut microbiome features were observed in male and female animals, as evidenced by the significant separation on the 3D-PCoA plot (A) (Red: Female; Blue: Male); Hierarchical clustering analysis by UPGMA indicates that male and female mice clustered in a gender-specific manner, with the UPGMA distance tree constructed at a distance of 0.1 (B).

Figure S2. Analysis at the Level 2 of Subsystem illustrate diazinon exposure altered the pathways of Amino acids and derivatives in a gender-selective manner. In male mice, the

pathways of *alanine, serine and glycine* and *aromatic amino acids and derivatives* were statistically significantly perturbed (A). In female mice, the pathway of *branched-chain amino acids* was altered (B). (* indicates p-value <0.05).

Figure S3. Analysis at the Level 3 of Subsystem illustrate diazinon exposure altered the pathways of *aromatic amino acids and derivatives* in a gender-selective manner. In male mice, the specific pathways of *aromatic amino acid degradation* and *Chorismate: intermediate for synthesis of Tryptophan* were statistically significantly perturbed (A). No statistical significance was observed in female mice (B). (* indicates p-value <0.05).

Figure S4. Serum metabolomic profiles were readily differentiated between the controls and diazinon-treated mice using principle component analysis (A: male mice; B: female mice). Pathway analysis using identified altered serum metabolites indicate that 7 and 5 metabolic pathways were significantly perturbed in male (C) and female animals (D) (p value <0.05), respectively. (Each dot represents one metabolic pathway; A metabolic pathway may not reach statistical significance if the number of metabolite hit in that specific pathway is too low; Only pathways with statistical significance are labeled herein).

Figure S5. Molecular features with p value <0.05 and fold change >1.5 were further filtered based on Variable Importance in Projection (VIP) scores associated with partial least squares discriminant analysis (PLS-DA) for the controls and treatment samples. Only features with VIP>1.8 were selected for identification with database search (A: male; B: female). These features are the most important ones to drive the separation between the controls and treatment samples.

Figure S6.The body weight gain was statistically significantly different between the controls and treated male mice after exposure to diazinon for 13 weeks (p=0.017 by Welch two sample t-test) (A); However, there was no statistically significant difference between controls and diazinon-treated female animals (p=0.24) (B). (All boxes extend from the 25th to the 75th percentile, horizontal bars represent the median, whiskers extend 1.5 times the length of the interquartile range (IQR) above and below the 75th and 25th percentiles, respectively, and outliers are represented as points).

Table S1. Relative abundance of typical gut bacterial genera in the measurements for control and diazinon exposed male mice[a].

Bacterial genus	Relative abundance (diazinon)	Standard error (diazinon)	Relative abundance (control)	Standard error (control)
Porphyromonadaceae_Tannerella*	0.015527766	0.001545025	4.02E-05	1.65E-05
Bacteroidetes_Other*	8.02E-05	3.53E-05	0.001177756	0.000124101
Bacteria_Other*	0.001552017	0.000294407	0.000246462	7.12E-05
Burkholderiales_Other*	0.001342627	0.000312732	0	0
Porphyromonadaceae_Other*	0.537398908	0.076928548	0.205295077	0.0291641
TM7_genera_incertae_sedis_Other*	0.000178574	5.09E-05	0	0
Lachnospiraceae_Johnsonella*	0.000586331	0.000133459	0.006825016	0.001879704
Lachnospiraceae_Shuttleworthia*	0	0	0.000132376	4.07E-05
Lachnospiraceae_Butyrivibrio*	0	0	0.000121039	3.88E-05
Bacteroidales_Other*	0.000572344	0.00018396	7.34E-06	7.34E-06
Porphyromonadaceae_Parabacteroides*	0.067233592	0.011232997	0.143951124	0.025158953
Staphylococcaceae_Staphylococcus*	0	0	2.94E-05	1.11E-05
Lachnospiraceae_Other*	0.084933938	0.03187199	0.23443195	0.046576008
Erysipelotrichaceae_Other*	0.000147949	0.000117405	0.000719056	0.000187227
Clostridiaceae_Other*	4.47E-05	3.41E-05	0	0
Erysipelotrichaceae_Allobaculum*	0.001171793	0.000321817	0.003862776	0.001059793
Lactobacillales_Other*	1.17E-05	1.17E-05	0.000179171	6.87E-05
Erysipelotrichaceae_Coprobacillus*	6.18E-05	2.60E-05	0	0
Bacteroidaceae_Bacteroides*	0.017143971	0.007316389	8.72E-06	8.72E-06
Lachnospiraceae_Hespellia	3.51E-05	3.51E-05	0.000468865	0.000197602
Ruminococcaceae_Other	0.013120116	0.004488096	0.031522134	0.007456963
Clostridiaceae_Clostridiaceae 1	0.016905914	0.004453174	0.061013566	0.020570682
Verrucomicrobiaceae_Akkermansia	0.002000862	0.000987381	0	0
Ruminococcaceae_Acetanaerobacterium	0.001370869	0.000364279	0.00361097	0.001135669
Erysipelotrichaceae_Erysipelotrichaceae Incertae Sedis	0	0	0.000185493	0.000102008
Incertae Sedis XIII_Anaerovorax	0.000285997	6.84E-05	0.000679013	0.000206743
Anaeroplasmataceae_Anaeroplasma	0.012968852	0.005038154	0.027610876	0.006710293
Enterobacteriaceae_Klebsiella	0	0	5.69E-05	3.45E-05
Ruminococcaceae_Ethanoligenens	3.36E-05	3.36E-05	0.000171765	8.04E-05
Lachnospiraceae_Lachnobacterium	0.000363653	0.000235776	0	0
Rikenellaceae_Alistipes	0.00013229	8.71E-05	0	0
Ruminococcaceae_Ruminococcus	0.002021593	0.001050989	0.005409658	0.001979717
Betaproteobacteria_Other	3.37E-05	3.37E-05	0	0
Erysipelotrichaceae_Bulleidia	1.68E-05	1.68E-05	0	0

Lachnospiraceae_Parasporobacterium	0.0040138	0.001142035	0.007020844	0.001764954
Ruminococcaceae_Ruminococcaceae Incertae Sedis	0.003187884	0.000709367	0.005097599	0.001361502
Lactobacillaceae_Lactobacillus	0.021096805	0.004506397	0.050446356	0.024051435
Lachnospiraceae_Lachnospira	0.002050337	0.000709393	0.005306687	0.002781304
Erysipelotrichaceae_Turicibacter	0.030454452	0.004442096	0.037431407	0.004647944
Ruminococcaceae_Sporobacter	0.0043351	0.001805655	0.006474919	0.001051172
Bradyrhizobiaceae_Afipia	1.69E-05	1.69E-05	0	0
Erysipelotrichaceae_Holdemania	3.59E-05	2.20E-05	3.08E-05	1.78E-05
Incertae Sedis XI_Anaerococcus	0	0	1.34E-05	1.34E-05
Lactobacillaceae_Other	0	0	3.22E-05	3.22E-05
Streptococcaceae_Streptococcus	0	0	1.34E-05	1.34E-05
Lachnospiraceae_Bryantella	0.004062191	0.002419823	0.006160614	0.001770821
Lachnospiraceae_Roseburia	0.001219102	0.000352915	0.001755485	0.000706761
Lachnospiraceae_Dorea	0.017511316	0.008339426	0.01221034	0.002203258
Eubacteriaceae_Anaerofustis	2.02E-05	1.26E-05	1.61E-05	1.61E-05
Ruminococcaceae_Faecalibacterium	2.93E-05	1.85E-05	7.34E-06	7.34E-06
Alphaproteobacteria_Other	6.33E-05	4.01E-05	3.88E-05	1.58E-05
Coriobacteriales_Coriobacterineae	0.000402264	0.000174141	0.000543562	0.000226359
Firmicutes_Other	0.00128476	0.000254072	0.001903292	0.001287543
Lachnospiraceae_Lachnospiraceae Incertae Sedis	0.067987204	0.021700208	0.07765687	0.002043561
Ruminococcaceae_Anaerotruncus	0.013359524	0.005809011	0.010550185	0.003940834
Clostridiaceae_Clostridiaceae 2	0.002095988	0.000392632	0.001906603	0.000481005
Clostridiales_Other	0.044532633	0.007597974	0.042497261	0.003420444
Ruminococcaceae_Papillibacter	0.004929792	0.001042412	0.005043531	0.001114149
Carnobacteriaceae_Carnobacteriaceae 2	0	0	8.72E-06	8.72E-06
Carnobacteriaceae_Other	0	0	6.69E-06	6.69E-06
Clostridia_Other	8.41E-06	8.41E-06	2.34E-05	1.53E-05
Enterococcaceae_Enterococcus	8.41E-06	8.41E-06	8.72E-06	8.72E-06
Flavobacteriaceae_Flavobacterium	1.69E-05	1.69E-05	7.34E-06	7.34E-06
Flexibacteraceae_Cardinium	0	0	7.34E-06	7.34E-06
Flexibacteraceae_Other	0	0	7.34E-06	7.34E-06
Streptophyta_Other	0	0	1.61E-05	1.61E-05

Relative abundance and standard error of individual bacterial genus were calculated by Metastats.

*: p value <0.05 when comparing the controls and diazinon-treated male mice.

[a] The "Other" assignments are typically due to ambiguity when the RDP classifier tries to assign at the desired level.

Table S2. Relative abundance of typical gut bacterial genera in the measurements for control and diazinon exposed female mice[a].

Bacterial genus	Relative abundance (diazinon)	Standard error (diazinon)	Relative abundance (control)	Standard error (control)
Clostridiaceae_Clostridiaceae 2*	0.003458619	0.000828862	0.012540825	0.001354591
Lachnospiraceae_Roseburia*	5.27E-05	2.21E-05	0.000209476	3.42E-05
Clostridiaceae_Clostridiaceae 1*	0.061952236	0.008370006	0.027014167	0.005424855
Lachnospiraceae_Johnsonella*	0.005963605	0.000740144	0.003478387	0.000164421
Porphyromonadaceae_Other*	0.399557821	0.015892862	0.326684115	0.016064827
Erysipelotrichaceae_Allobaculum*	0.001525694	0.000653572	0.004798136	0.000846603
Ruminococcaceae_Other*	0.016573418	0.001562632	0.024547067	0.002259338
Clostridiales_Other*	0.026358609	0.004103712	0.044492961	0.00565986
Lachnospiraceae_Other*	0.07158608	0.009091446	0.097595947	0.004501038
Ruminococcaceae_Ruminococcus*	0.001392252	0.000439532	0.008777964	0.002855036
Incertae Sedis XIII_Anaerovorax*	0.000415317	7.71E-05	0.000905692	0.000177361
Lachnospiraceae_Lachnospira*	0.002010958	0.000774436	0.006660515	0.001821575
Erysipelotrichaceae_Erysipelotrichaceae Incertae Sedis*	2.37E-05	1.70E-05	0.000180352	6.54E-05
Clostridia_Other	2.03E-05	1.39E-05	6.45E-05	1.60E-05
Bacteria_Other	0.000319041	8.30E-05	0.000529718	5.90E-05
Firmicutes_Other	0.001802876	0.000398207	0.005570144	0.002038302
Streptococcaceae_Streptococcus	6.30E-06	6.30E-06	6.22E-05	3.21E-05
Clostridiaceae_Other	0.000181914	6.06E-05	0.000384979	0.000113314
Bacteroidales_Other	0.000126501	5.04E-05	4.62E-05	1.29E-05
Ruminococcaceae_Anaerotruncus	0.000592008	0.000161404	0.000876504	9.37E-05
Staphylococcaceae_Staphylococcus	0	0	3.81E-05	3.04E-05
Ruminococcaceae_Sporobacter	0.00583425	0.000978362	0.004172091	0.001051322
Erysipelotrichaceae_Coprobacillus	0	0	2.69E-05	1.20E-05
Lactobacillales_Other	5.95E-05	4.14E-05	0.000146385	6.69E-05
Erysipelotrichaceae_Turicibacter	0.070406973	0.007343036	0.087488662	0.014208233
Enterobacteriaceae_Klebsiella	1.40E-05	1.40E-05	0	0
Porphyromonadaceae_Parabacteroides	0.147112334	0.0112601	0.158722372	0.005178616
Lachnospiraceae_Acetitomaculum	0.00080638	0.00021865	0.000553528	0.00017136
Ruminococcaceae_Acetanaerobacterium	0.00201382	0.000186682	0.002509007	0.000591777
Lactobacillaceae_Lactobacillus	0.030946907	0.003726459	0.035545119	0.005252514
Bacteroidetes_Other	0.00094184	0.000272568	0.001139627	7.49E-05
Anaplasmataceae_Wolbachia	0	0	1.35E-05	1.35E-05
Lactobacillaceae_Other	0	0	1.43E-05	8.81E-06
Streptophyta_Other	0	0	1.30E-05	7.97E-06

Lachnospiraceae_Shuttleworthia	0	0	2.22E-05	9.21E-06
Ruminococcaceae_Ruminococcaceae Incertae Sedis	0.004661264	0.000389768	0.005350086	0.001174142
Bacilli_Other	2.03E-05	1.39E-05	1.77E-05	1.10E-05
Ruminococcaceae_Ethanoligenens	1.97E-05	1.97E-05	3.12E-05	2.42E-05
Lachnospiraceae_Dorea	0.07943636	0.005017129	0.073098108	0.012352289
Coriobacteriales_Coriobacterineae	0.000648983	0.000269001	0.000523992	7.58E-05
TM7_genera_incertae_sedis_Other	4.34E-05	2.11E-05	5.91E-05	3.06E-05
Lachnospiraceae_Lachnospiraceae Incertae Sedis	0.034079006	0.00335465	0.036490779	0.004764343
Erysipelotrichaceae_Holdemania	3.23E-05	2.06E-05	2.89E-05	1.90E-05
Lachnospiraceae_Parasporobacterium	0.004107562	0.000788197	0.00443112	0.000477824
Ruminococcaceae_Papillibacter	0.002112699	0.00027156	0.002223404	0.000240653
Erysipelotrichaceae_Other	0.000479939	0.000127357	0.000547672	0.000190107
Anaeroplasmataceae_Anaeroplasma	0.019291342	0.002700468	0.018417645	0.002890365
Lachnospiraceae_Bryantella	0.002415938	0.000337069	0.002328933	0.000317619
Proteobacteria_Other	0.000136773	5.27E-05	0.00012022	7.44E-05
Lachnospiraceae_Hespellia	0.000390392	0.000249756	0.000433064	0.000133148
Carnobacteriaceae_Other	0	0	8.33E-06	8.33E-06
Lachnospiraceae_Oribacterium	0	0	1.01E-05	1.01E-05
Actinobacteria_Other	3.06E-05	3.06E-05	8.33E-06	8.33E-06
Bacillaceae_Bacillaceae 2	0	0	6.73E-06	6.73E-06
Moraxellaceae_Acinetobacter	0	0	8.33E-06	8.33E-06
Porphyromonadaceae_Tannerella	3.71E-05	2.28E-05	2.34E-05	1.63E-05
Pseudomonadaceae_Other	0	0	8.33E-06	8.33E-06

Relative abundance and standard error of individual bacterial genus were calculated by Metastats.

*: p value <0.05 when comparing the controls and diazinon-treated female mice.

[a] The "Other" assignments are typically due to ambiguity when the RDP classifier tries to assign at the desired level.

Table S3. Identified significantly changed fecal metabolites (fold change >1.5 p < 0.05 compared to controls) in male mice

Name	Fold	p value	Mass	Retention time	Gut flora related	Pathways or functions
Taurine	-106.15	0.001	326.20	18.52	Y	Bile Acid Biosynthesis
Glyceric acid	4.66	0.016	232.20	13.27		Glycerolipid Metabolism
L-Aspartic acid	-4.41	0.015	235.20	14.86		Aspartate Metabolism
Lithocholic acid	4.33	0.010	427.40	35.12	Y	Bile Acid Biosynthesis
5-Hydroxyindoleacetic acid	4.15	0.045	409.30	24.92	Y	Tryptophan Metabolism
Glycine	-3.17	0.038	249.20	12.85		Alanine Metabolism
alpha-Ketoglutaric acid	-2.74	0.015	302.20	17.19		Citric Acid Cycle
L-Proline	-2.57	0.049	175.20	12.73		Arginine and Proline Metabolism
Stigmastanol	2.54	0.010	237.20	34.95	Y	Steroids and Steroid Derivatives
2-Oleoylglycerol	2.48	0.028	107.20	30.41	Y	
Hydrocinnamic acid	-2.39	0.003	132.10	14.81	Y	phenylalanine metabolism
Octadecanoic acid	-2.24	0.018	359.30	25.76		Fatty acid metabolism
Butanedioic acid	-2.22	0.019	150.10	13.01	Y	Citric Acid Cycle
5-Aminovaleric acid	-2.20	0.011	266.20	17.95		Amino acid biosynthesis
L-Isoleucine	-2.18	0.027	114.10	12.62		Leucine and Isoleucine [illegible]
DL-Phenylalanine	-2.13	0.041	65.10	16.84	Y	Phenylalanine/Tyrosine [illegible]
Campesterol	2.09	0.013	177.20	35.00	Y	Steroids and Steroid Derivatives
L-Threonine	-1.97	0.037	146.20	12.66		Glycine and Serine Metabolism
(Hydroxyphenyl)propanoic acid	-1.96	0.009	311.20	19.42	Y	Phenols and Derivatives
Linoleic acid	1.95	0.014	245.20	25.34		Linoleic Acid Metabolism
Glycerol 3-phosphate	1.89	0.035	274.20	19.80		Glycerolipid Metabolism
Stigmasterol	1.87	0.024	76.10	35.21	Y	Steroids and Steroid Derivatives
Cholesterol	1.79	0.047	299.30	34.16	Y	Bile Acid Biosynthesis
L-Glutamic acid	-1.77	0.026	130.10	17.86		Amino acid metabolism
3-Hydroxypyridine	-1.77	0.009	64.10	8.01		Propanoate Metabolism
L-Valine	-1.72	0.007	113.10	11.24		Phenols and Derivatives
4-Hydroxyphenylacetic acid	-1.69	0.043	78.10	18.16	Y	Tyrosine Metabolism
Phenylacetic acid	-1.52	0.040	75.10	12.76	Y	Phenylacetic Acid Derivatives
L-Alanine	-1.66	0.046	70.10	9.18		Alanine Metabolism
Uracil	-1.64	0.007	127.10	13.39		Beta-Alanine Metabolism
(Methoxyimino)propanoic acid	-1.63	0.037	115.10	8.20		
Palmitic acid	-1.60	0.045	317.30	23.48		Fatty Acid Biosynthesis

Table S4. Identified significantly changed fecal metabolites (fold change >1.5 $p < 0.05$ compared to controls) in female mice.

Name	Fold	p value	Mass	Retention Time	Gut flora related	Pathways or functions
l-Aspartic acid	15.70	0.020	207.20	14.85		Amino Acids and Derivatives
Lithocholic acid	5.09	0.004	592.50	35.10	Y	Bile Acid Biosynthesis
5beta-Cholestanone	-5.09	0.030	328.40	33.42	Y	Cholesterols and Derivatives
(Hydroxyphenyl)propanoic acid	-3.89	0.023	311.20	19.41	Y	Phenols and Derivatives
Deoxycholic acid	3.62	0.001	253.30	35.21	Y	Bile Acid Biosynthesis
Stigmastanol	-3.03	0.017	488.60	34.94	Y	Steroids and Steroid Derivatives
L-Alanine	2.75	0.008	101.10	9.19		Amino Acids and Derivatives
2-Oleoylglycerol	-2.53	0.016	67.20	33.40	Y	
3-Glycerophosphate	-2.48	0.024	117.10	19.80		Glycerolipid Metabolism
Carbamic acid	2.13	0.017	205.10	7.81		Amino Acids and Derivatives
Stigmasterol	2.05	0.010	184.20	35.20	Y	Steroids and Steroid Derivatives
5alpha-Cholestanol	-2.02	0.034	258.30	34.26	Y	Cholesterols and Derivatives
Cholesterol	-2.01	0.049	371.40	34.18	Y	Bile Acid Biosynthesis
Behenic acid	-1.89	0.032	353.40	29.71		Fatty Acids and Conjugates
Tetracosanoic acid	-1.67	0.001	381.40	31.50		Fatty Acids metabolism
Hydrocinnamic acid	1.61	0.014	65.10	14.79		phenylalanine metabolism
Lactic acid	1.53	0.019	107.10	8.42	Y	Pyruvate Metabolism
Fumaric acid	1.51	0.011	144.15	13.64	Y	Citrate cycle

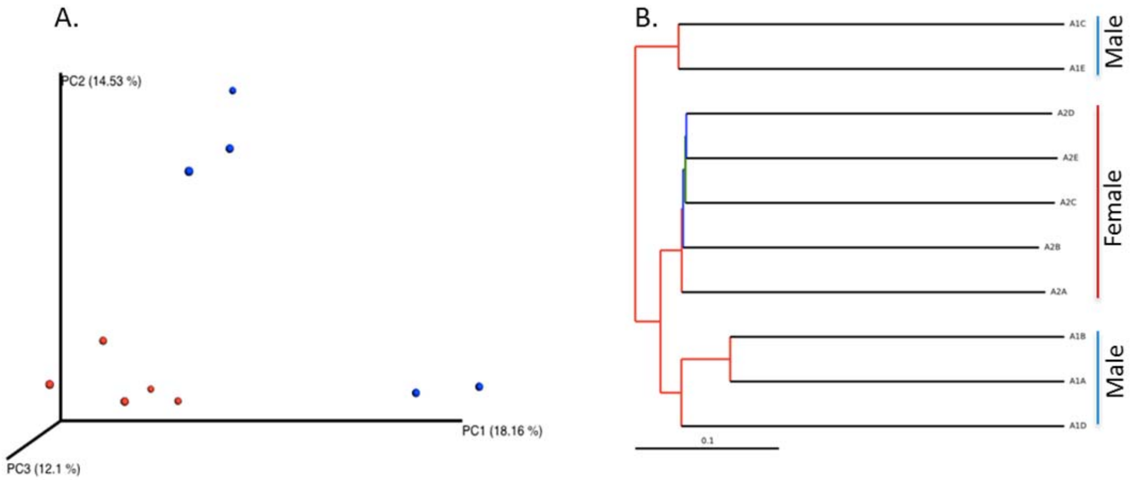

Figure S1. Distinct gut microbiome features were observed in male and female animals, as evidenced by the significant separation on the 3D-PCoA plot (A) (Red: Female; Blue: Male); Hierarchical clustering analysis by UPGMA indicates that male and female mice clustered in a gender-specific manner, with the UPGMA distance tree constructed at a distance of 0.1 (B).

Leve 2 analysis (Amino acids and derivatives)

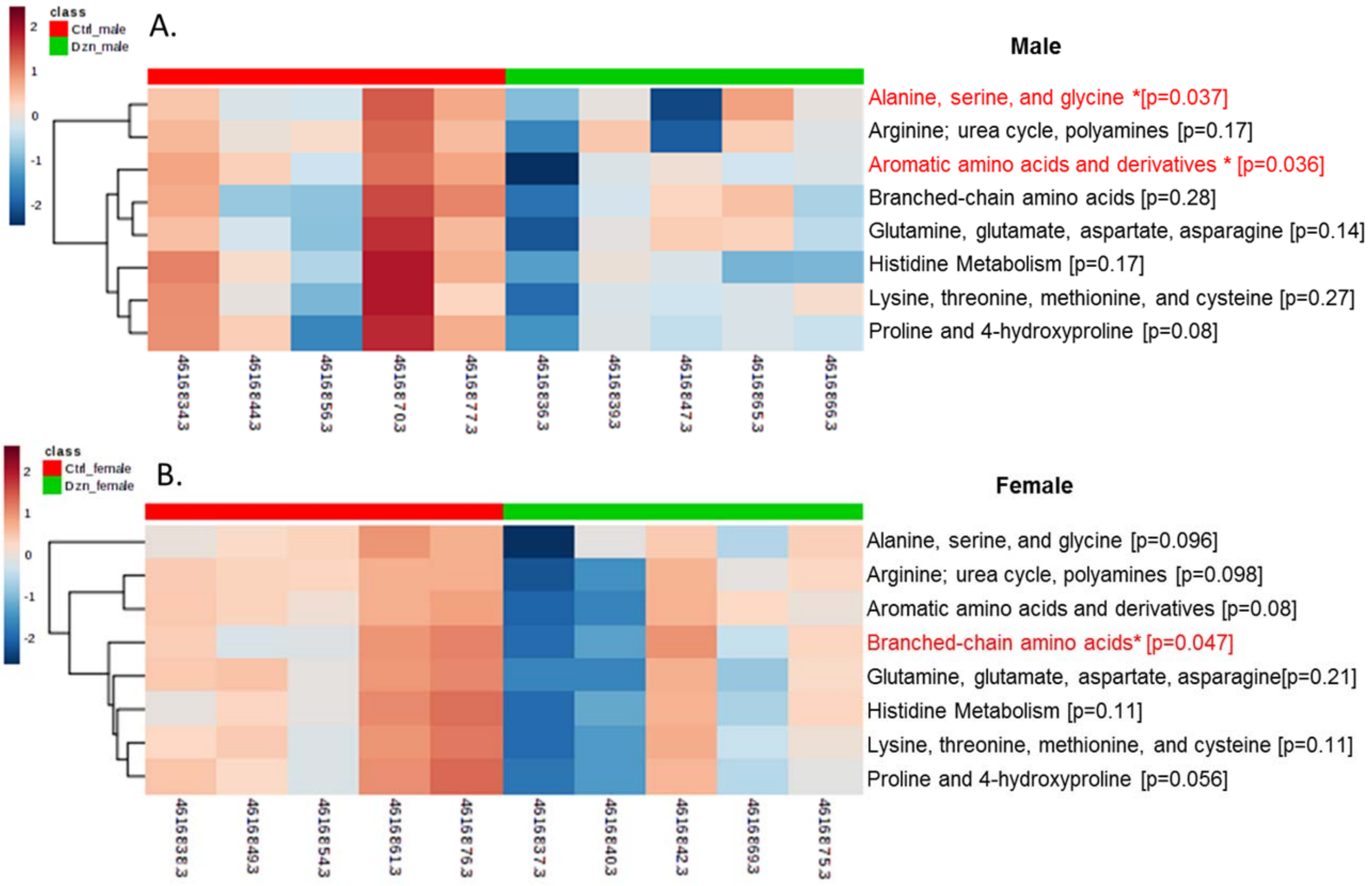

Figure S2. Analysis at the Level 2 of Subsystem illustrate diazinon exposure altered the pathways of Amino acids and derivatives in a gender-selective manner. In male mice, the pathways of *alanine, serine and glycine* and *aromatic amino acids and derivatives* were statistically significantly perturbed (A). In female mice, the pathway of *branched-chain amino acids* was altered (B). (* indicates p-value <0.05).

Leve 3 analysis (Aromatic amino acids and derivatives)

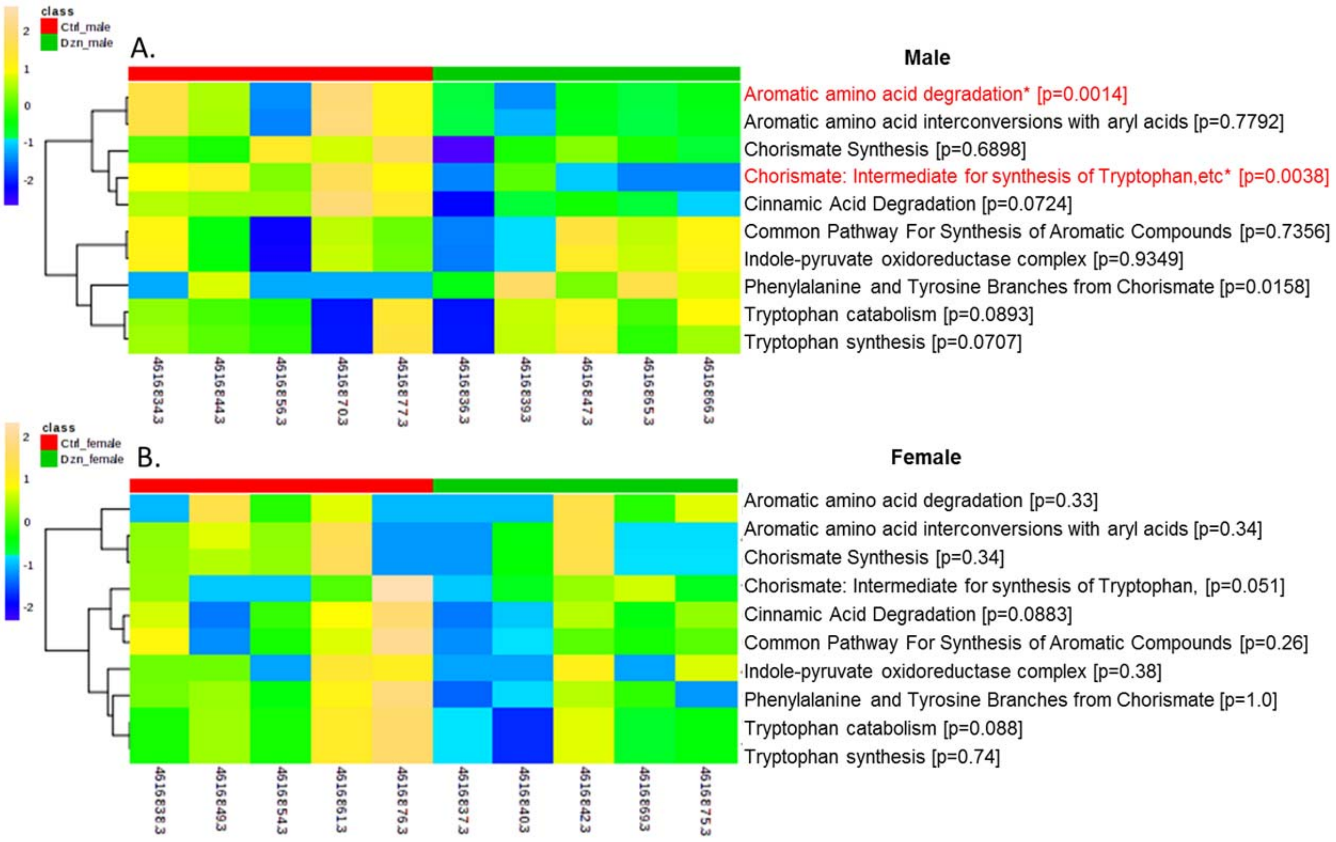

Figure S3. Analysis at the Level 3 of Subsystem illustrate diazinon exposure altered the pathways of *aromatic amino acids and derivatives* in a gender-selective manner. In male mice, the specific pathways of *aromatic amino acid degradation* and *Chorismate: intermediate for synthesis of Tryptophan* were statistically significantly perturbed (A). No statistical significance was observed in female mice (B). (* indicates p-value <0.05).

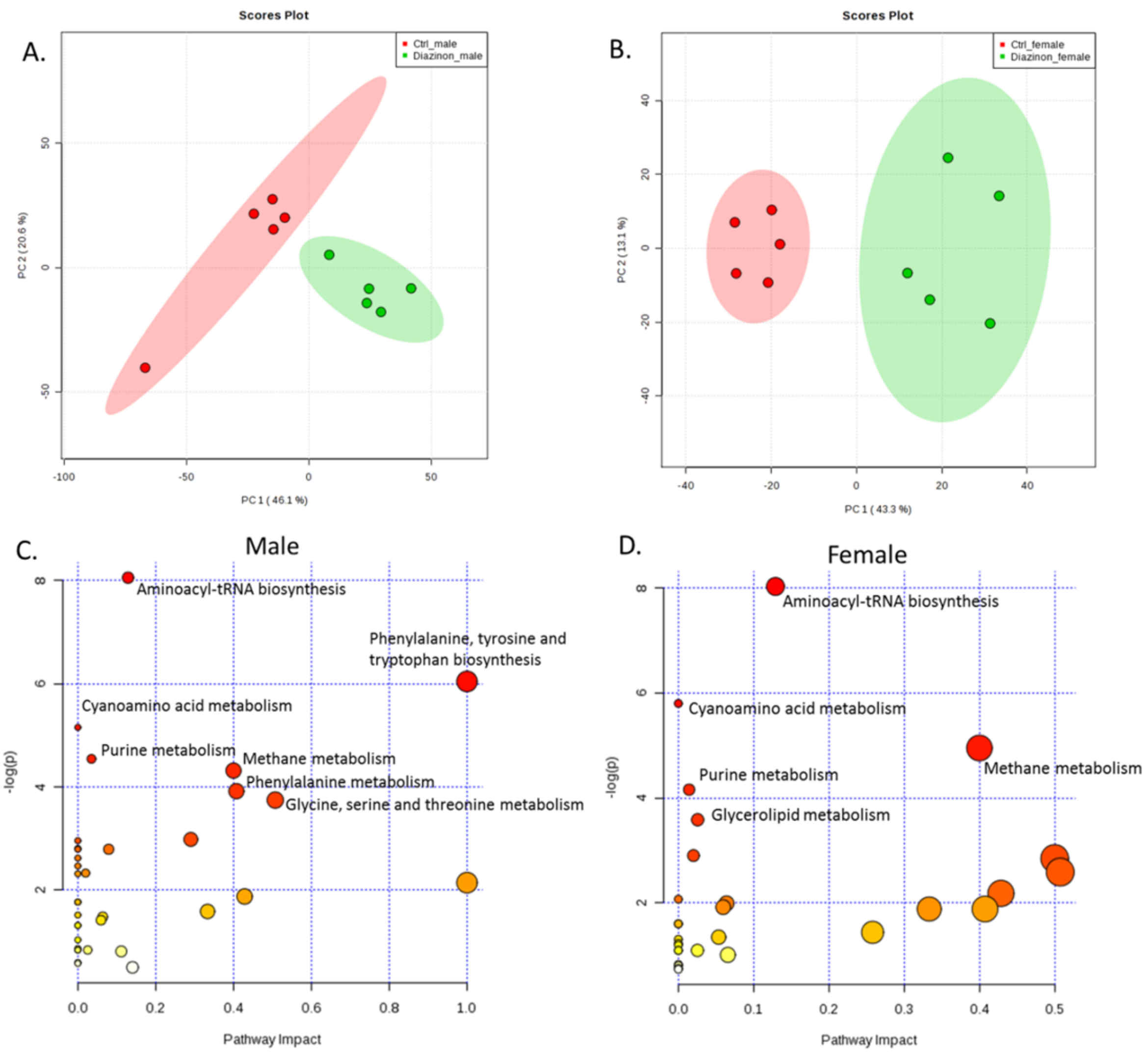

Figure S4. Serum metabolomic profiles were readily differentiated between the controls and diazinon-treated mice using principle component analysis (A: male mice; B: female mice). Pathway analysis using identified altered serum metabolites indicate that 7 and 5 metabolic pathways were significantly perturbed in male (C) and female animals (D) (p value <0.05), respectively. (Each dot represents one metabolic pathway; A metabolic pathway may not reach statistical significance if the number of metabolite hit in that specific pathway is too low; Only pathways with statistical significance are labeled herein).

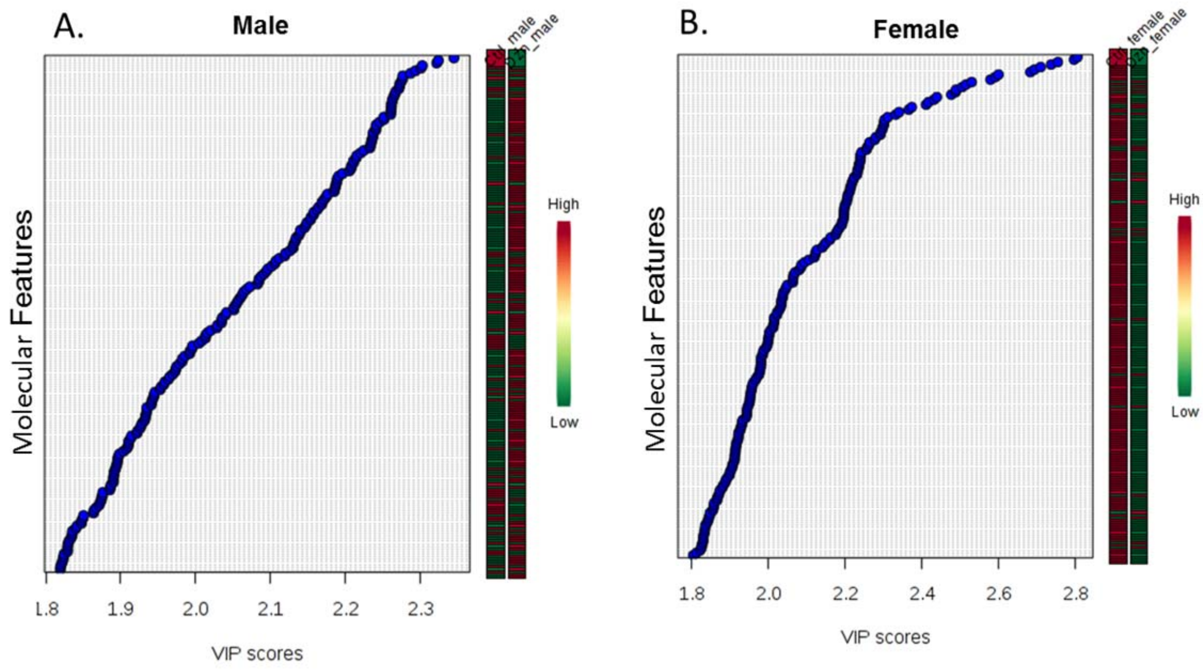

Figure S5. Molecular features with p value <0.05 and fold change >1.5 were further filtered based on Variable Importance in Projection (VIP) scores associated with partial least squares discriminant analysis (PLS-DA) for the controls and treatment samples. Only features with VIP>1.8 were selected for identification with database search (A: male; B: female). These features are the most important ones to drive the separation between the controls and treatment samples.

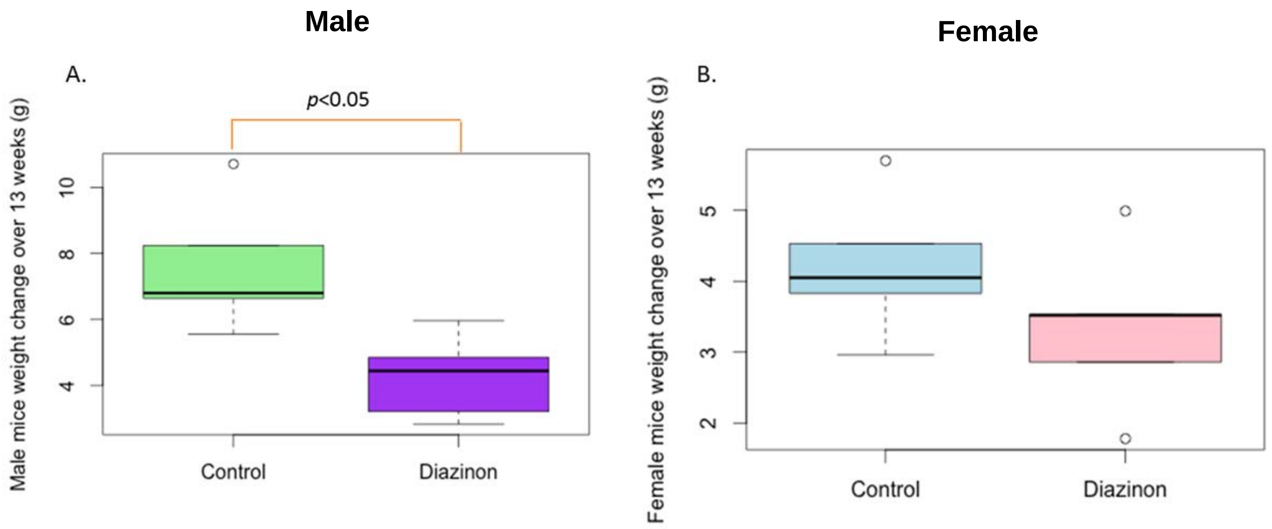

Figure S6.The body weight gain was statistically significantly different between the controls and treated male mice after exposure to diazinon for 13 weeks (p=0.017 by Welch two sample t-test) (A); However, there was no statistically significant difference between controls and diazinon-treated female animals (p=0.24) (B). (All boxes extend from the 25th to the 75th percentile, horizontal bars represent the median, whiskers extend 1.5 times the length of the interquartile range (IQR) above and below the 75th and 25th percentiles, respectively, and outliers are represented as points).
